# Efficient 3D late gadolinium enhancement imaging using the CLAWS respiratory motion control algorithm

**DOI:** 10.1186/1532-429X-15-S1-O63

**Published:** 2013-01-30

**Authors:** Jennifer Keegan, Permi Jhooti, Sonya V Babu-Narayan, Peter Drivas, Sabine Ernst, David N Firmin

**Affiliations:** 1 Cardiovascular Biomedical Research Unit, Royal Brompton and Harefield NHS Trust, London, UK; 2 Cardiology, Royal Brompton and Harefield NHS Trust, London, UK; 3 Imperial College of Science Technology and Medicine, London, UK

## Background

Acquisition durations of navigator gated high resolution 3D late gadolinium enhancement (LGE) studies are long (1,2). While implementation of the continuously adaptive windowing strategy (CLAWS (3)) - which results in the fastest possible acquisition duration for a given breathing pattern and navigator acceptance window size - may be beneficial, the respiratory-dependent and therefore, non-smooth k-space acquisition order during gadolinium wash-out could result in increased image artifact. This study was performed to investigate if CLAWS could be used to increase the respiratory efficiency of 3D LGE imaging without detriment to image quality.

## Methods

Whole-heart 3D (32-36 slices, 1.5 x 1.5 x 4 mm, reconstructed to 64-72 slices, 0.7 x 0.7x.2 mm) inversion-prepared segmented gradient echo imaging was performed in 18 consecutive patients on a Siemens 1.5 Tesla Avanto scanner. Two acquisitions were performed in random order, one with CLAWS respiratory motion control and one with an end-expiratory tracking accept/reject algorithm (EE-ARA). Imaging started 15 minutes post-gadolinium administration with inversion-time scouting both before and after each acquisition. Paired t-testing was used to compare the acquisition durations against the best possible scan times that could have been achieved for the patient-specific respiratory patterns which were determined from retrospective analysis of the navigator data stored with each acquisition. CLAWS and EE-ARA qualitative image quality scores (ranging from 1 = non-diagnostic to 5 = excellent) were compared using paired-Wilcoxon analysis.

## Results

**Figure 1:**
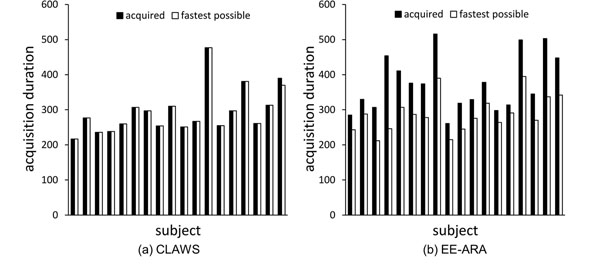
Acquisition durations in cardiac cycles (black) compared with the fastest possible acquisition durations (white) in 18 subjects for CLAWS (a) and EE-ARA (b) respiratory motion control. The fastest possible acquisition durations are determined from retrospective analysis of the navigator data stored with each acquisition.

**Figure 2:**
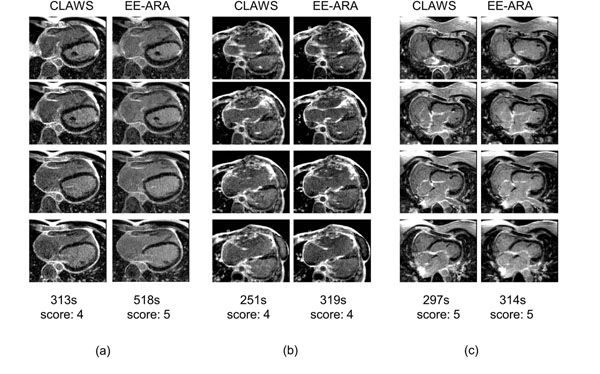
Four selected slices from example CLAWS (left) and EE-ARA (right) datasets in three subjects ((a), (b), (c)), together with acquisition durations and image quality scores.

Retrospective analysis of the navigator data stored with the acquisitions shows that CLAWS results in scan times which are very close to (within 1%) or equal to the fastest achievable scan times (293.8 +/- 65.0 cardiac cycles vs 292.7 +/- 63.4 cardiac cycles, p = ns) while EE-ARA significantly extends the scan duration (374.8 +/- 81.4 cardiac cycles vs 289.2 +/- 51.4 cardiac cycles, p <.0001) (see Figure 1). EE-ARA acquisitions are 26% longer than CLAWS acquisitions (378 +/- 104 s vs 301 +/- 85 s, p = .002). Image quality scores are slightly but not significantly reduced for CLAWS scans (4.1 +/- 0.6 vs 4.3 +/- 0.6, p = ns). Example data is shown in Figure 2. Detailed numerical phantom simulations confirm that for noise levels consistent with in vivo acquisitions, the non-uniform k-space ordering introduced by the CLAWS algorithm does not result in changes in blood signal-to-noise ratio (21.4 +/- 4.0 vs 22.9 +/- 4.8, p = ns) or in blood-myocardium contrast to noise ratio (19.3 +/- 3.9 vs 19.9 +/- 4.5, p = ns).

## Conclusions

We conclude that the CLAWS algorithm allows efficient acquisition of free-breathing 3D LGE without detriment to the image quality.

## Funding

Wellcome Trust: WT093953MA; NIHR (National Institute for Health Research); British Heart Foundation Intermediate Clinical Research Fellowship.
